# Physiology of Sea-Sickness

**Published:** 1853-10

**Authors:** 


### *The Physiology of Sea-sickness.*

By Marshall Hall, F. R. S. (Virginia Med. and Surgical Journal from Comptes Rendus.) I have recently made a voyage from Liverpool to the United States, and I seized upon this occasion to study the physiology of sea-sickness.

All of the phenomena of this disease induce me to believe that the spinal cord is the nervous centre, and that the pneumo-gastric, diaphragmatic, intercostal, and abdominal nerves, are the eisodic and exodic nerves which, in connexion with this centre, present the origin and course of the catastaltic and diastaltic actions, in these circumstances.

It appeared to me that the rolling and pitching of the ship influenced especially the course of the blood in the spinal cord; when the vessel rises, the force of the impulse of the blood upon this organ is diminished; when it falls, the impulse is augmented. There is then constant change in the force of this impulse; from which results perpetual excitement and irritation of the cord, of the pneumo-gastric and diaphragmatic nerves, etc.

The movement of a carriage or of a swing, if they are long continued produce the same effects upon very susceptible persons.

The influence of the position of the body, by which the movements of elevation or depression are increased or diminished, is very remarkable. If a horizontal position in the direction of the axis of movement of the vessel is selected and is preserved, the traveler may escape sea-sickness, and it is only upon changing this position that he suffers the first symptoms.

At first he experiences an indescribable uneasiness, a faintness at the stomach: presently acidity, eructation, hiccough supervenes; these symptoms being produced by the influence of the pneumo-gastric and diaphragmatic nerves upon the secretions and movements of the stomach, of the diaphragm, etc. Almost simultaneously the patient becomes pale, and the action of the heart becomes feeble and irregular, and sometimes palpitations occur; this also is an affection of the pneumo-gastric nerve. One of my friends suffered for years from irregular action of the heart, after experiencing violent sea-sickness during a voyage of a few hours. Lastly, nausea occurs, and distressing vomiting; the stomach is first emptied, and afterwards bile, mucus, etc. are ejected.

These phenomena are repeated by paroxysms; a suffocating sensation of heat compels the individual to throw off the clothing, the warmth of which had been previously agreeable; then vomiting takes place, the pores of the skin are opened and a cold perspiration occurs, which is followed by a sensation of chillness. It is impossible to convey any idea of the complete prostration that the patient experiences during an attack of sea-sickness, both morally and physically. In one case, that of a young lady, this cruel disease terminated in death.

Before and after the paroxysms, the action of the pneumo-gastric nerve is defective; its influence as an external excitor of the respiration is imperfect; this state is relieved by the free exposure of the face, and even of the hands, to the open air, and especially to the fresh wind, and by strong voluntary efforts of respiration.

If the sea becomes calm, one becomes habituated to a slight movement, but if a storm arises, he is presently sick again. I had enjoyed six days of physical comfort, when we experienced the effects of a gale; I suffered from sea-sickness for forty-eight hours.

